# Clinical outcomes of patients hospitalized for COVID-19 versus SARS: a meta-analysis

**DOI:** 10.18632/aging.104139

**Published:** 2020-11-24

**Authors:** Po-Cheng Chang, Chien-Chang Yang, Kuo-Chin Kao, Ming-Shien Wen

**Affiliations:** 1 Division of Cardiology, Department of Internal Medicine, Chang Gung Memorial Hospital, Linkou and Chang Gung University, Medical School, Taoyuan, Taiwan; 2 Division of Infectious Diseases, Department of Internal Medicine, Chang Gung Memorial Hospital, Linkou and Chang Gung University, Medical School, Taoyuan, Taiwan; 3 Division of Thoracic Medicine, Department of Internal Medicine, Chang Gung Memorial Hospital, Linkou and Chang Gung University, Medical School, Taoyuan, Taiwan

**Keywords:** COVID-19, severe acute respiratory syndrome (SARS), fatality

## Abstract

Estimating the case-fatality rate and clinical outcomes for patients with coronavirus disease 2019 (COVID-19) is crucial because health care systems must adequately prepare for outbreaks and design appropriate policies. A systematic search of PubMed, Embase, and Medline+Journal (via OVID) were conducted for relevant journal publications from database inception to May 4, 2020. Articles that reported the fatality rates and clinical outcomes of patients hospitalized for COVID-19 or severe acute respiratory syndrome (SARS) infection were included. Nine clinical reports (four SARS reports and five COVID-19 reports) with a total of 851 patients (367 and 484 patients with SARS and COVID-19, respectively) were analyzed. A greater proportion of hospitalized patients with COVID-19 had bilateral pneumonia (90.0% [76.3%–96.2%] vs. 35.9% [21.4%–53.6%], p < 0.001) and required ventilators (23.8% [18.8%–29.6%] vs. 15.3% [11.9%–19.4%], p = 0.010) compared with hospitalized patients with SARS. The case-fatality rate was 9.5% (6.5%–13.7%) and 6.1% (3.5%–10.3%) among patients with COVID-19 and SARS, respectively (p = 0.186). The case-fatality rate among hospitalized patients with COVID-19 was comparable to that during the 2003 SARS outbreak. A higher incidence of bilateral pneumonia and increased ventilator usage were noted among patients with COVID-19 compared with patients with SARS.

## INTRODUCTION

The ongoing coronavirus disease 2019 (COVID-19) pandemic is caused by SARS-CoV-2. The 2003 outbreak of severe acute respiratory syndrome (SARS) and the 2012 outbreak of Middle East respiratory syndrome were also caused by coronaviruses. The COVID-19 [1] and SARS outbreaks [2] were both first reported in China, and they had similar clinical presentations. How the case-fatality rates of COVID-19 and SARS infections compare is a matter of ongoing debate. The total case-fatality rate among patients with SARS in 2003 was 9.6%, and the case-fatality rate was 15.3% among patients in countries other than China [3]. Early reports revealed a 5%–16% case-fatality rate among patients hospitalized for COVID-19 pneumonia [4–8]. However, a report from the Chinese Center for Disease Control and Prevention estimated an overall case-fatality rate of 2.3% [9]. Some reports have implied that COVID-19 infection has a lower case-fatality rate than SARS infection [10, 11].

Estimating case-fatality rates is crucial because health care systems must adequately prepare for outbreaks and implement appropriate policies. However, determining the case-fatality rate during the COVID-19 pandemic is challenging. In particular, real-time report databases and clinical observation without adequate follow-up during the outbreak may provide misleading information. During an overwhelming outbreak, shortages of diagnostic tests and therapeutic resources can result in distorted outcome data. Accurate records of symptoms and presentations are essential for estimating outcomes. Therefore, we considered that medical data acquired from hospitalized patients may satisfy the fundamental requirements for accuracy.

The objective of this study was to compare clinical outcomes in patients hospitalized for COVID-19 with those of patients hospitalized for SARS. This study assessed case-fatality rate, pulmonary infiltration in chest radiography, pneumonia over the bilateral lungs, progression to acute respiratory distress syndrome (ARDS), ventilator use, and intensive care unit (ICU) admission rate.

## RESULTS

**Figure 1:**
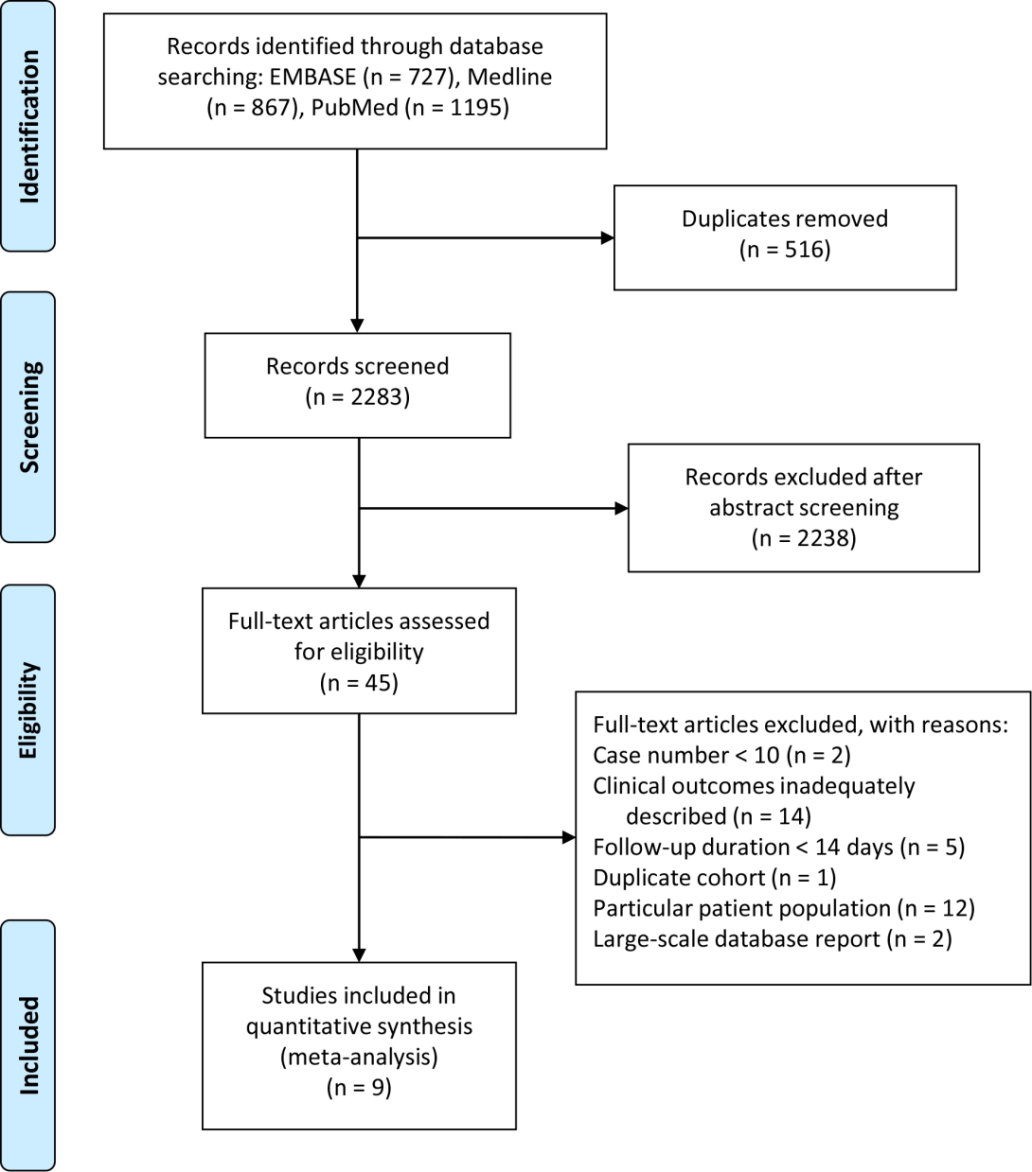
**Study flow (PRISMA) diagram.**

A literature search yielded a total of 1372 records, from which we removed 669 duplicates (Figure 1). After abstract screening, we excluded 660 studies that did not fulfill the inclusion criteria. After assessing the full text of 45 articles, we included 4 SARS articles (for a total of 367 patients, published between March 2003 and May 2003) and 5 COVID-19 articles (484 patients, published between January 2020 and March 2020). Table 1 shows the included studies and the main outcomes. In these papers, the diagnostic criteria for SARS were defined by the United States Centers for Disease Control and Prevention and the World Health Organization (WHO) [3]. As the time of the SARS outbreak, reverse-transcriptase polymerase chain reaction (RT-PCR) was not yet being widely applied for obtaining diagnostic confirmation, and only 20 patients with SARS received the RT-PCR test retrospectively. In the COVID-19 papers, the diagnostic criteria for COVID-19 infection included a positive SARS-CoV-2 RT-PCR test as specified by WHO interim guidance [12].

**Table 1:** Studies of patients hospitalized with SARS and COVID-19.

	**Location**	**Diseases**	**N**	**Bilateral or multiple pneumonia**	**Ventilator use**	**Death**
**Booth CM. et al, 2003 [25]**	Canada	SARS	144	75 (52%)	20 (14%)	8 (6%)
**Tsang KW. et al, 2003 [28]**	Hong Kong	SARS	10			2 (20%)
**Lee N. et al, 2003 [26]**	Hong Kong	SARS	138	49 (36%)	19 (14%)	5 (4%)
**Peiris JS. et al, 2003 [27]**	Hong Kong	SARS	75	16 (21%)	15 (20%)	5 (7%)
**Huang C. et al, 2020 [5]**	China	COVID-19	41	40 (98%)	14 (34%)	6 (15%)
**Liu K. et al, 2020 [8]**	China	COVID-19	137	116 (85%)	34 (25%)	16 (12%)
**Chen N. et al, 2020 [4]**	China	COVID-19	99	74 (75%)	17 (17%)	11 (11%)
**Wang D. et al, 2020 [6]**	China	COVID-19	138	138 (100%)	32 (23%)	6 (4%)
**Wang Z. et al, 2020 [7]**	China	COVID-19	69			5 (7%)

### Patient characteristics and clinical presentation

Demographics, comorbidities, and clinical presentations are listed in Table 2. Among the included articles, the 484 patients with COVID-19 were older than the 367 patients with SARS (53.0 ± 4.9 years and 42.0 ± 3.2 years, respectively, p < 0.001). There were more male patients in the COVID-19 group than in the SARS group (54.8% and 44.1%, p < 0.001). The COVID-19 group had a higher prevalence of chronic diseases than the SARS group did, such as cardiovascular diseases (17.4% and 5.8%, p < 0.001), hypertension (17.2% and 10.0%, p = 0.018), diabetes (10.2% and 7.9%, p < 0.001), and chronic obstructive pulmonary disease (COPD) (2.9% and 1.7%, p < 0.001). More patients with COVID-19 presented with cough, sputum production, and malaise than patients with SARS; however, fever, myalgia, headache, dizziness, sore throat, nausea or vomiting, and diarrhea were more common in patients with SARS than in patients with COVID-19. Patients with COVID-19 had a higher level of B-type natriuretic peptide (BNP) than patients with SARS. However, patients with SARS had a higher level of creatinine and alanine transaminase (ALT) than did patients with COVID-19.

**Table 2:** Demographics, comorbidities, and clinical presentations of patients with COVID-19 and SARS

**Variable**	**COVID-19**		**SARS**		
	**Valid *n***	**Data**	**Valid *n***	**Data**	***P* value**
Demographics					
Age, year	484	53.0 ± 4.9	367	42.0 ± 3.2	<0.001
Male	484	265 (54.8)	367	162 (44.1)	<0.001
Comorbidity					
Cardiovascular disease	484	84 (17.4)	292	17 (5.8)	<0.001
Hypertension	443	76 (17.2)	10	1 (10.0)	0.018
Chronic liver disease	248	6 (2.4)	213	10 (4.7)	<0.001
Diabetes	344	35 (10.2)	292	23 (7.9)	<0.001
Chronic obstructive pulmonary disease	385	11 (2.9)	292	5 (1.7)	<0.001
Clinical presentation					
Fever	484	430 (88.8)	367	366 (99.7)	<0.001
Myalgia	484	142 (29.3)	367	211 (57.5)	<0.001
Cough	415	260 (62.7)	367	209 (56.9)	<0.001
Sputum	344	63 (18.3)	292	48 (16.4)	0.043
Malaise	275	140 (50.9)	144	45 (31.3)	<0.001
Dizziness	207	18 (8.7)	357	68 (19.0)	<0.001
Headache	484	43 (8.9)	367	146 (39.8)	<0.001
Sore throat	306	35 (11.4)	367	61 (16.6)	<0.001
Nausea or vomiting	306	9 (2.9)	292	56 (19.2)	<0.001
Diarrhea	484	38 (7.9)	357	62 (17.4)	<0.001
Lab data					
Hemoglobin, g/dL	209	12.9 ± 0.2	223	13.4 ± 0.1	<0.001
Platelet count, ×1,000/μL	168	196.0 ± 21.0	367	168.0 ± 16.2	<0.001
White blood count, /μL	347	5422 ± 1469	367	5439 ± 541	0.832
Neutrophil, /μL	347	3678 ± 1115	282	3747 ± 150	0.302
Lymphocyte, /μL	347	898 ± 133	292	900	0.810
Creatinine, mg/dL	347	0.89 ± 0.23	367	0.99 ± 0.11	<0.001
BUN, md/dL	237	14.1 ± 2.1	282	12.7 ± 1.6	<0.001
Bilirubin total, mg/dL	140	0.82 ± 0.09	357	0.86 ± 0.72	0.540
ALT, U/L	347	29.4 ± 6.5	367	47.5 ± 14.9	<0.001
BNP, pg/mL	237	14.1 ± 2.1	282	12.7 ± 1.6	<0.001

Data are presented as a number (percentage) or the mean ± standard deviation.

ALT, alanine transaminase; BUN, blood urea nitrogen; BNP, B-type natriuretic peptide.

### Outcome analyses

**Figure 2:**
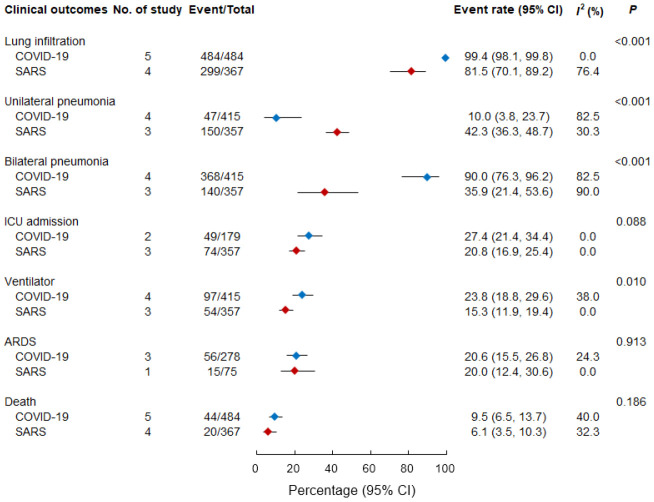
**Pooled event rate of clinical outcomes in patients with COVID-19 and SARS.** ARDS, acute respiratory distress syndrome; ICU, intensive care unit; SARS, severe acute respiratory syndrome.

Figure 2 presents a comparison of clinical outcomes between study groups. Hospitalized patients with COVID-19 and SARS frequently developed pneumonia. Among the hospitalized patients, a higher percentage of those with COVID-19 had an initial presentation of lung infiltration (99.4% [98.1%–99.8%]) than those with SARS (81.5% [70.1%–89.2%], p < 0.001). Patients with COVID-19 had a higher likelihood (90.0% [76.3%–96.2%]) of developing bilateral pneumonia than patients with SARS (35.9% [21.4%–53.6%], p < 0.001).

Although the data concerning ICU admission were limited, the rate of ICU admission was higher among patients with COVID-19 (27.4% [21.4–34.4%]) than among those with SARS (20.8% [16.9%–25.4%]; p = 0.088). A notably higher proportion of patients with COVID-19 required a mechanical ventilator (23.8% [18.8%–29.6%]) than that of patients with SARS (15.3% [11.9%–19.4%]; p = 0.010). However, the proportions of patients who progressed to ARDS were comparable between patients with COVID-19 (20.6% [15.5%–26.8%] and patients with SARS (20.0% [12.4%–30.6%]; p = 0.913). As indicated in Figure 2, high case-fatality rates were observed both for hospitalized patients with COVID-19 (9.5% [6.5%–13.7%]) and hospitalized patients with SARS (6.1% [3.5%–10.3%]; p = 0.186).

Supplementary Figures 1–12 show funnel plots for the assessment of publication bias. Egger’s tests indicated no significance, with the exception of the results for lung infiltration from the COVID-19 studies (p = 0.002). Analyses of publication bias could not be performed for ICU admission rates from the COVID-19 studies nor for ARDS from the SARS studies because fewer than three studies were available.

## DISCUSSIONS

In the included articles, patients hospitalized for COVID-19 had a higher average age than those hospitalized for SARS. The prevalence of cardiovascular disease, hypertension, diabetes, and COPD among patients with COVID-19 was also higher than that among patients with SARS. Although reports showed a high prevalence of asymptomatic carriers or cases with minimal symptoms among patients with COVID-19, COVID-19 infection was associated with a higher rate of abnormal chest radiography findings than was SARS infection. Hospitalized patients with COVID-19 also had a greater risk of developing bilateral pneumonia and a higher prevalence of progression to ARDS compared with patients hospitalized for SARS infection. The case-fatality rates among patients hospitalized for COVID-19 and SARS infection were similarly high.

### Clinical presentations

Clinical presentations did not include the loss of smell or loss of taste as symptoms in the eligible articles. Researchers have recently become aware of olfactory and gustatory disorders as prevalent symptoms among patients with COVID-19, who may not have other airway symptoms [13]. Whether patients with SARS also developed loss of smell or loss of taste is unclear. Notably, anosmia was found in one case reported during the SARS outbreak [14].

Clinical manifestations are not the only indicators of active virus shedding. Although most patients included in this study were symptomatic, asymptomatic COVID-19 and SARS infections and colonizations were also present [15–17]. Asymptomatic patients were able to spread the virus to other members of the public. Moreover, some patients could spread the virus even after recovering from COVID-19 or SARS infection [18, 19]. Asymptomatic carriers or patients with few symptoms may pose problems for containing virus outbreaks. Therefore, wearing a facemask could be helpful for containment in light of silent spreaders.

### High prevalence of cardiovascular disease, hypertension, and diabetes

The COVID-19 group had a higher prevalence of cardiovascular disease, hypertension, and diabetes than the SARS group. SARS-CoV-2, like other coronaviruses, utilizes spike glycoprotein to bind to angiotensin-converting enzyme 2 (ACE2) on the host cell surface [20]. One report showed that the affinity of SARS-CoV-2 to human ACE2 is higher than that of SARS-CoV [21]. The use of ACE inhibitors and angiotensin-converting enzyme blockers (ARBs) increases cellular expression of ACE2 [22]. This finding may partially explain the higher prevalence of cardiovascular disease, hypertension, and diabetes in the COVID-19 group than in the SARS group. Increased ACE2 expression raises the concern of using ACE inhibitors or ARBs in patients with COVID-19 infection. However, adequate evidence to confirm the causal association between the use of ACE inhibitors or ARBs and a higher risk of COVID-19 infection is lacking. A recent report indicated that the use of ACE inhibitors and ARBs was more common among patients with COVID-19 than among controls because of their higher prevalence of cardiovascular disease rather than because of their use of these medications [23].

### Variation of outcome estimation

Estimation of case outcomes may vary by country because of differences in mitigation policies, diagnostic criteria, extent of RT-PCR tests, and availability of health care. Patients commonly remain asymptomatic or have mild symptoms after COVID-19 or SARS infection. Many patients without severe symptoms were not counted as confirmed cases. In one RT-PCR study, approximately 11.5% of well-protected health care workers exposed to patients with SARS were found to have virus colonization or infection, and only approximately 10% of these developed severe symptoms [16]. Another report also revealed that 78% of cases identified during the COVID-19 outbreak were asymptomatic [15]. The policies for RT-PCR swab tests vary among countries: some conduct extensive testing, and some provide testing only for symptomatic cases. These findings demonstrate that accurate estimation is a challenge. However, many fatal cases may go unrecognized because they did not fulfill the testing criteria or because health care institutions are unable to provide testing during overwhelming outbreaks. These variations mean that estimates of case fatality in large-scale patient populations are unreliable. Therefore, we assessed patient outcomes of a hospitalized population from reports by using data observed directly in hospitals.

From a public health perspective, accurate clinical outcome estimates are essential for officials to make adequate preparations and implement appropriate policies for virus containment. As of April 12, 2020, Taiwan had a total of 388 confirmed COVID-19 cases, including only 54 locally transmitted cases. The case number was far fewer than anticipated, with initial models predicting that Taiwan would be at the second-highest risk of imported cases [24]. Initial reports of the COVID-19 outbreak in Wuhan, China [4–8] revealed a similar clinical pattern to that of the SARS outbreak in 2003 [25–28]. Early precautions and awareness of the disease’s severity played a prominent role in Taiwan’s success in containing COVID-19 [29].

### Limitations

The diagnostic criteria for SARS and COVID-19 were different. The COVID-19 reports included patients who fulfilled the WHO diagnostic criteria and had positive RT-PCR test results. However, the diagnostic criteria for SARS were based on clinical presentation and history of direct contact with a person who became ill after exposure to an index patient.

The patient populations were also different: the COVID-19 group was older, had more male patients, and had a higher prevalence of chronic diseases than the SARS group did. The poorer outcomes among patients with COVID-19 may be associated with their older age and higher prevalence of chronic diseases.

These two outbreaks occurred in different countries and regions. The case-fatality rate is substantially affected by the preparedness and availability of health care services. Health care systems have advanced in the past two decades, and most institutions were able to manage ARDS. The fatality rate may increase during fast-spreading periods, and the reports analyzed in this study were published before overwhelming COVID-19 or SARS outbreaks.

Patients with mild symptoms were not included in this study; therefore, the study lacks a direct comparison of severity scores between patients who were hospitalized for COVID-19 and those hospitalized for SARS. We do not reject the possibility of different severities between these two groups because the existing data are insufficient to measure the severity of pneumonia. The reports included only hospitalized patients, and thus patients with mild symptoms were not included. During the 2003 SARS outbreak, some of these cases of mild infection were also undetected because of limited molecular diagnostic tools. In an RT-PCR study, approximately 11.5% of well-protected health care workers exposed to patients with SARS were found to have virus colonization or infection, and only approximately 10% of these developed severe symptoms [16]. According to epidemiological reports [30], positive anti–SARS-CoV immunoglobin G seroprevalence was 0.23% among SARS health care workers and 0.10% among healthy individuals, indicating the existence of undiagnosed patients with SARS with relatively mild symptoms. During the early stages of the SARS and COVID-19 outbreaks, physicians admitted patients on the basis of clinical judgment rather than positive molecular test results. Therefore, the severity of pneumonia in the case studies should indicate the fulfillment of criteria for admission.

## CONCLUSIONS

In summary, the patients hospitalized for COVID-19 were older and had a higher prevalence of chronic diseases than patients hospitalized for SARS. COVID-19 infection was associated with a higher rate of abnormal chest radiography findings, a greater risk of developing bilateral pneumonia, and a higher incidence of progression to ARDS compared with SARS infection. The case-fatality rate was similarly high among patients hospitalized for COVID-19 and SARS infections.

## MATERIALS AND METHODS

### Search strategy and selection criteria

This meta-analysis of published studies was conducted following the Preferred Reporting Items for Systematic Reviews and Meta-Analyses (PRISMA) guidelines. A systematic search of PubMed, Embase (via Ovid), and Medline journals (via Ovid) was conducted. The database search period was from database inception to May 4, 2020. The complete search algorithms are provided in the supplementary data. The keywords included a combination of severe acute respiratory syndrome, SARS, SARS-CoV-2, COVID-19, coronavirus, and case report (see Supplementary Material 1). Only full-text English-language journal articles and research involving humans were included. Two researchers (Chang PC and Yang CC) evaluated the included studies by using the United States National Heart, Lung, and Blood Institute and Research Triangle Institute Study Quality Assessment (Supplementary Table 1). All included studies were assessed for quality. The two researchers independently extracted clinical presentation and outcome data. The data were reevaluated, and consensus was reached in the case of discrepancies.

### Eligibility criteria

All clinical reports that included clinical outcomes (including chest radiography presentations, ventilator support, ICU admission, and death) for COVID-19 and SARS infection were included. Case reports or case series with fewer than 10 patients were excluded. Papers reporting only laboratory outcomes, radiology presentations, or epidemiological information rather than detailed clinical outcomes were also excluded. Because clinical outcomes are time dependent, papers were also excluded if the follow-up duration of the enrolled patients was less than 14 days. We did not include studies that enrolled only particular patient populations, such as pregnant women, fatal cases, patients undergoing hemodialysis, or asymptomatic cases. If papers were based on the same group of patients, we included the article with the largest patient group and the most complete outcome data. Because the disease severity in hospital-based direct observation studies was discrepant with data from large-scale database reports, this study included only hospital-based direct observation studies (Figure 1).

### Data extraction

Relevant information was collected by Chang PC and Yang CC. The study-level characteristics extracted were the name of first author, publication year, region, number of patients, patient demographics (age and sex), 6 types of comorbidity, 10 categories of clinical presentation, and 10 types of laboratory results (if available). Data for outcomes of interest were collected, including abnormal chest radiography results, unilateral pneumonia, bilateral pneumonia, ICU admission, progression to ARDS, ventilator usage, and death.

### Data analysis

This meta-analysis included studies that did not directly compare the study groups (COVID-19 and SARS) and pooled rates of events from the included studies for comparison. Random-effects models were used to pool the estimates (the rates of events) of clinical outcomes from individual studies for each study group. An *I*^2^ value greater than 50% was considered to indicate substantial heterogeneity across the studies. The pooled estimates between COVID-19 and SARS were compared using mixed-effects models. Statistical significance was set at a two-tailed p value of <0.05. Publication bias was assessed using a visual check of the funnel plot together with Egger’s test. Data were analyzed using Comprehensive Meta-Analysis software (version 3; Biostat, Englewood, NJ, USA).

## Supplementary Material

Supplementary Figures

Supplementary Table 1

Supplementary Material
