# Supplementary material for: Clinical outcomes of patients hospitalized for COVID-19 versus SARS: a meta-analysis
Source: Aging (Albany NY). 2020 Nov 24;12(24):24552–69. doi: 10.18632/aging.104139 (PMC7803544; doi:10.18632/aging.104139)
Supplement: Supplementary Figures [file aging-12-104139-s001.pdf]

SUPPLEMENTARY FIGURES

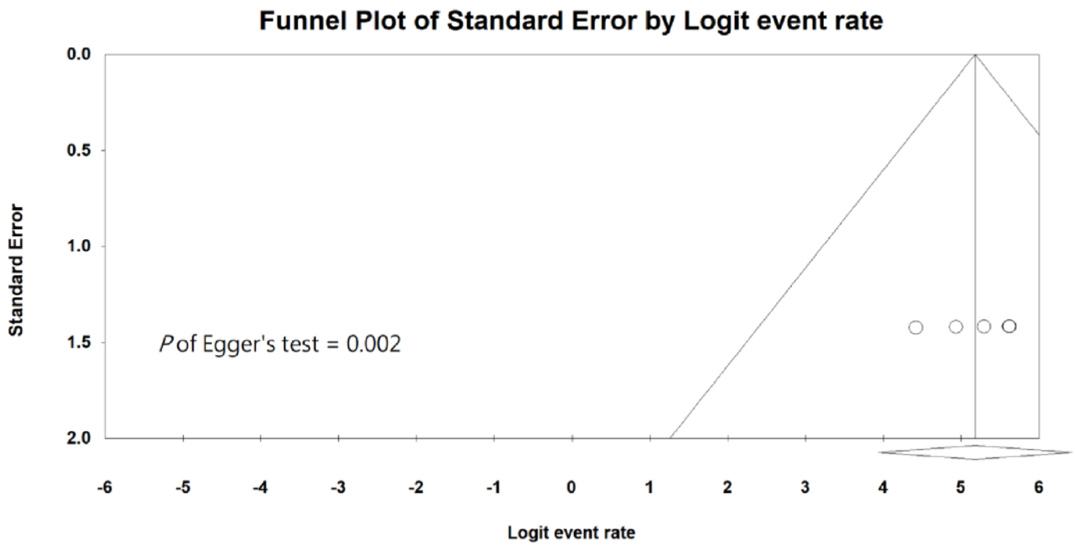

Supplementary Figure 1. Funnel plot for assessment of publication bias on lung infiltration from COVID-19 studies.

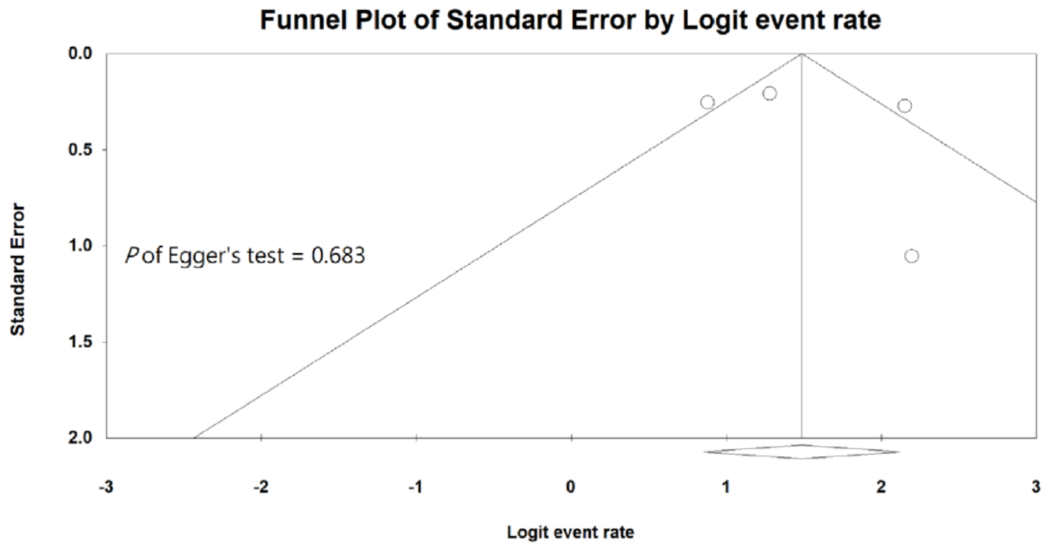

Supplementary Figure 2. Funnel plot for assessment of publication bias on lung infiltration from SARS studies.

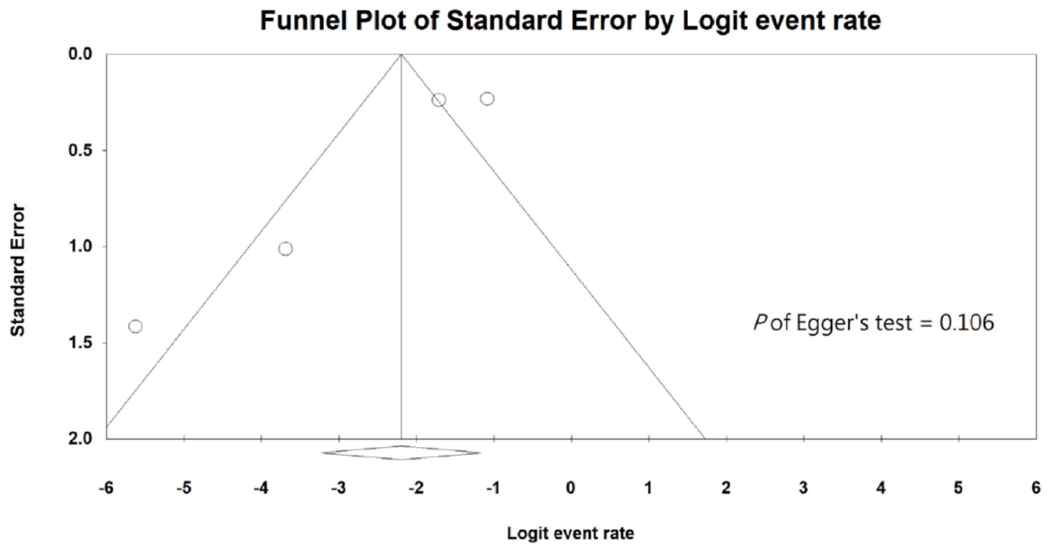

Supplementary Figure 3. Funnel plot for assessment of publication bias on unilateral pneumonia from COVID-19 studies.

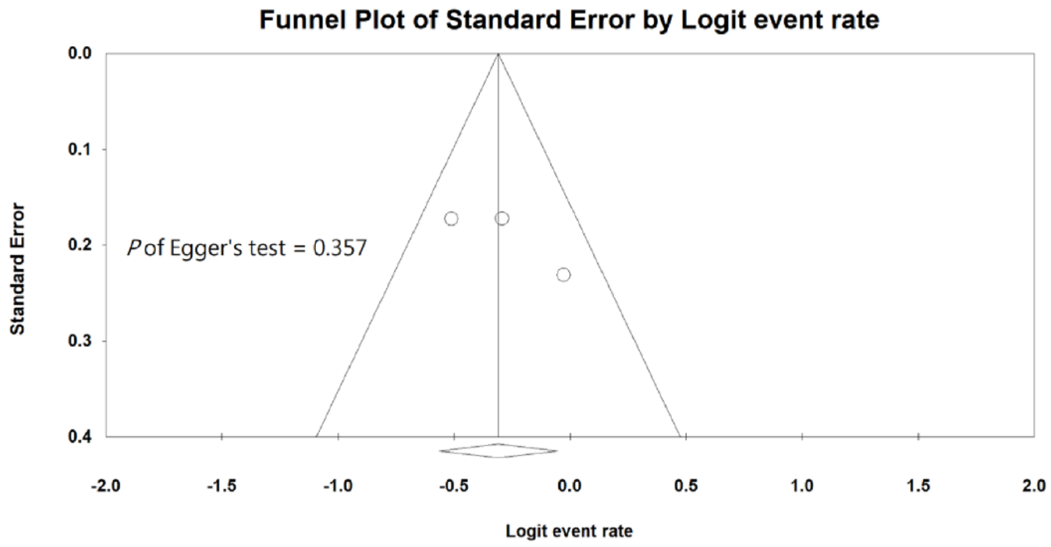

Supplementary Figure 4. Funnel plot for assessment of publication bias on unilateral pneumonia from SARS studies.

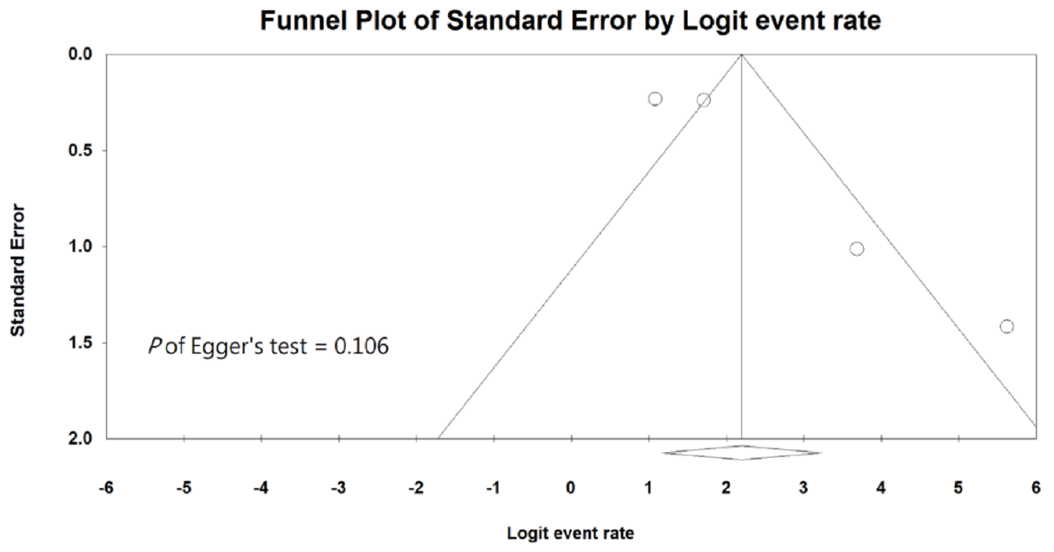

Supplementary Figure 5. Funnel plot for assessment of publication bias on bilateral pneumonia from COVID-19 studies.

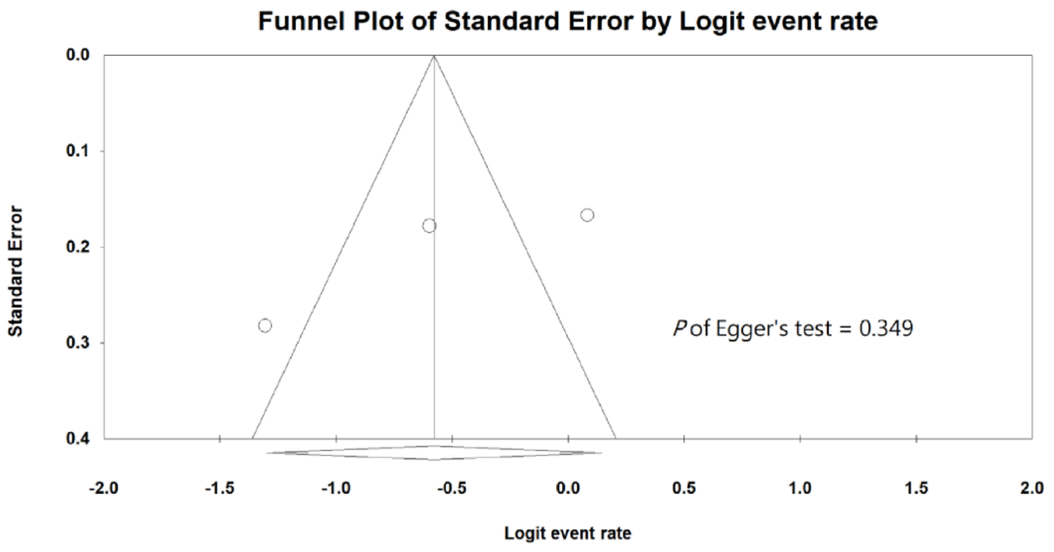

Supplementary Figure 6. Funnel plot for assessment of publication bias on bilateral pneumonia from SARS studies.

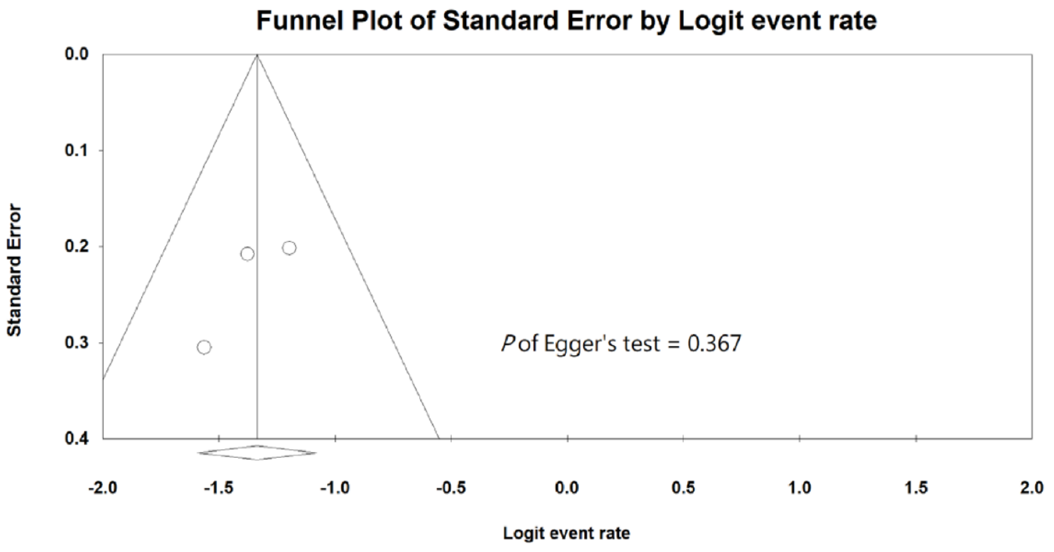

Supplementary Figure 7. Funnel plot for assessment of publication bias on ICU admission from SARS studies.

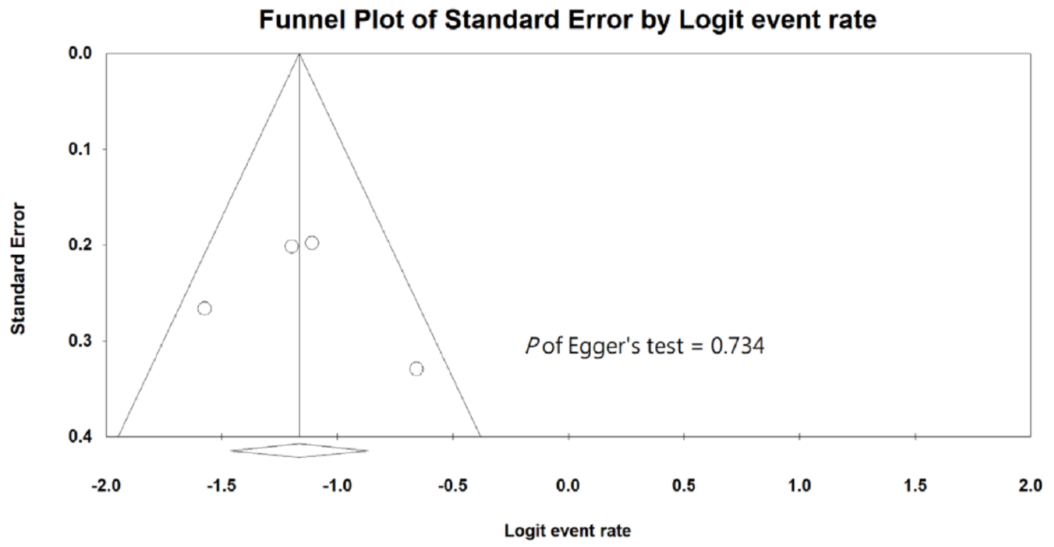

Supplementary Figure 8. Funnel plot for assessment of publication bias on ventilation from COVID-19 studies.

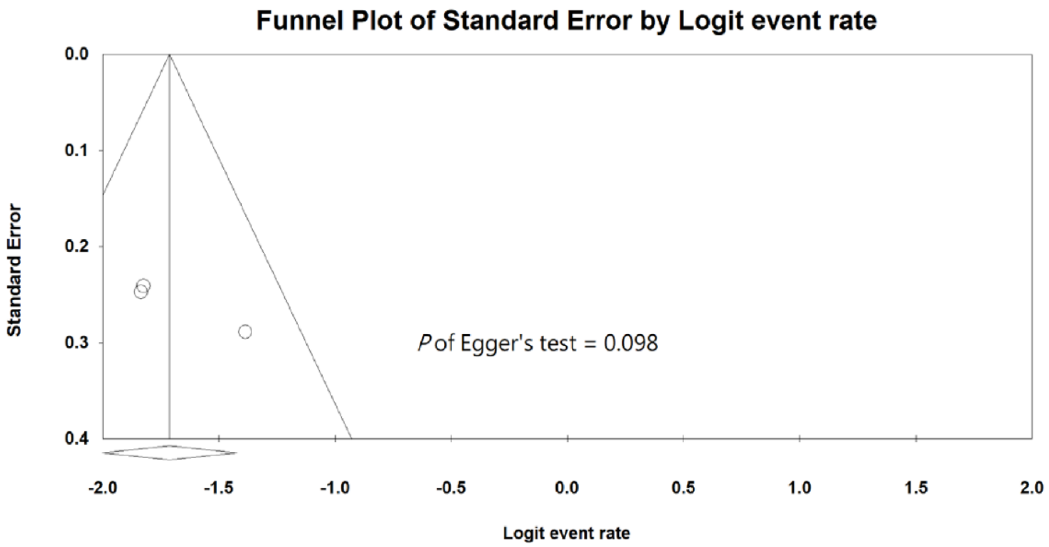

Supplementary Figure 9. Funnel plot for assessment of publication bias on ventilation from SARS studies.

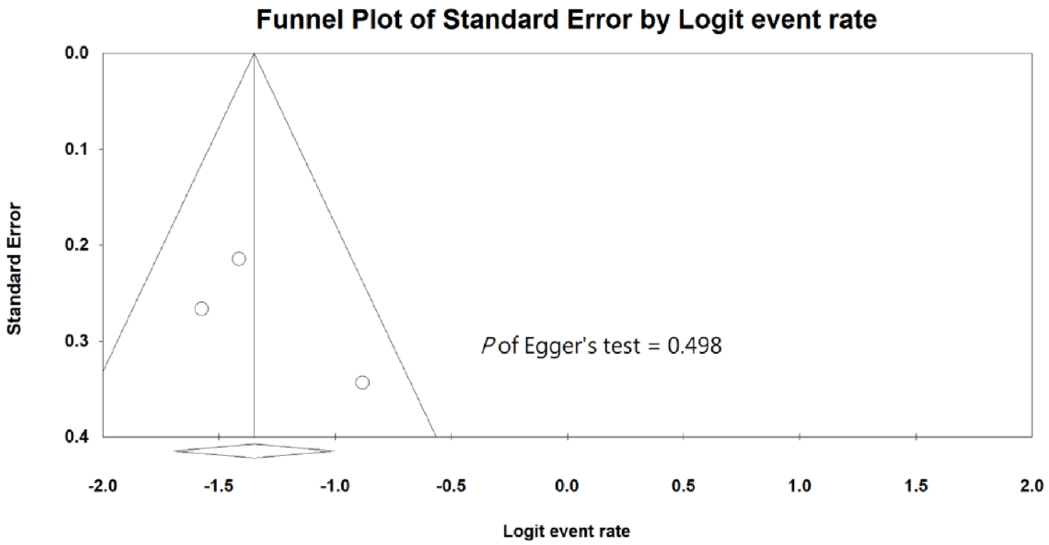

Supplementary Figure 10. Funnel plot for assessment of publication bias on ARDS from COVID-19 studies.

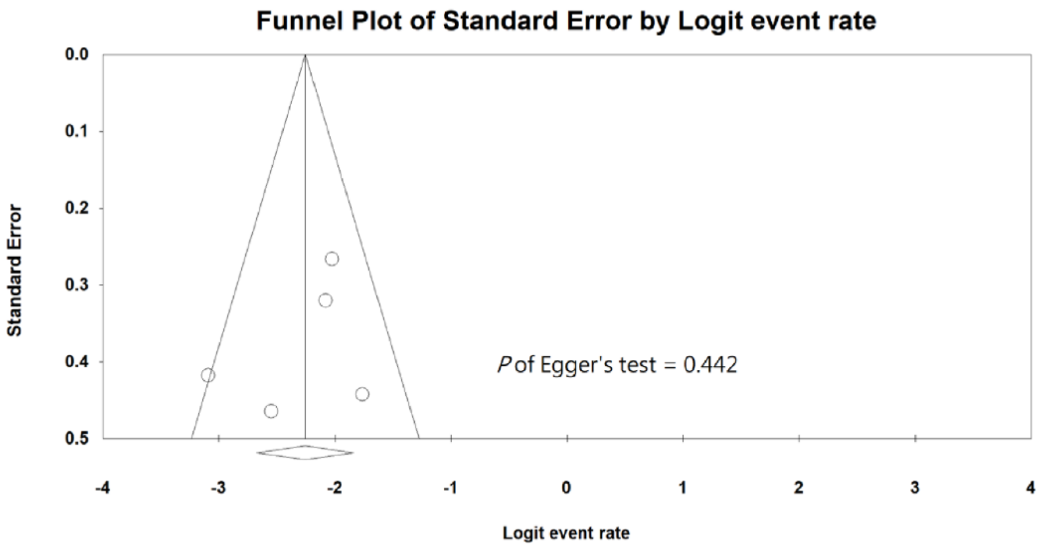

Supplementary Figure 11. Funnel plot for assessment of publication bias on death from COVID-19 studies.

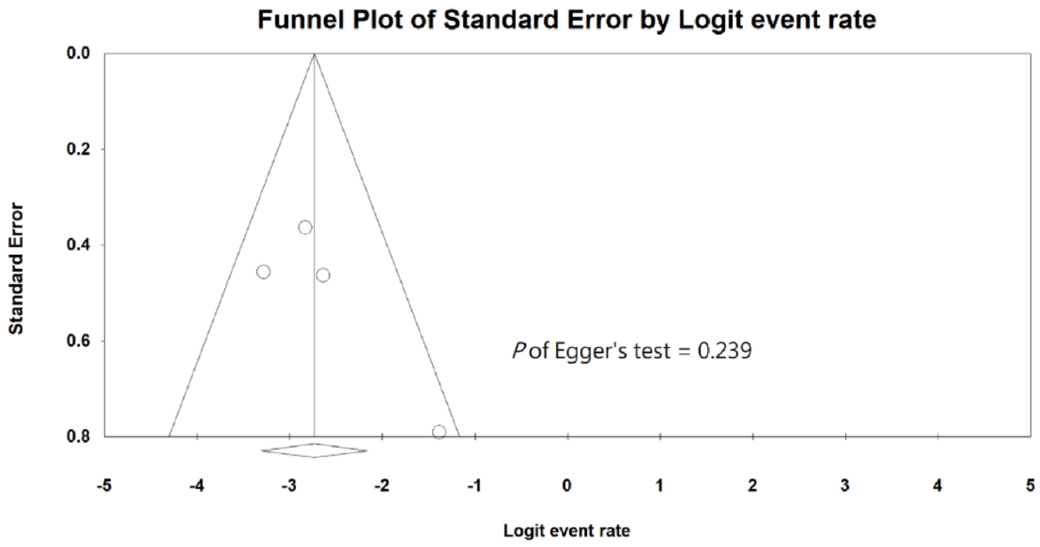

Supplementary Figure 12. Funnel plot for assessment of publication bias on ARDS from SARS studies.
