# Supplementary material for: Clinical outcomes of patients hospitalized for COVID-19 versus SARS: a meta-analysis
Source: Aging (Albany NY). 2020 Nov 24;12(24):24552–69. doi: 10.18632/aging.104139 (PMC7803544; doi:10.18632/aging.104139)
Supplement: Supplementary Material [file aging-12-104139-s003.pdf]

## Supplementary Material

**Supplementary Material 1. The exact string of keywords used to do the search in each database.**

Embase:
('severe acute respiratory distress syndrome' OR 'sars'/exp OR '2019-ncov' OR 'covid 19'/exp) AND ('case report'/de OR 'clinical article'/de) AND 'human'/de AND 'article'/it

Medline+Journals@Ovid
((severe acute respiratory syndrome or SARS or SARS-CoV-2 or COVID-19) and coronavirus and cases).mp. [mp=title, abstract, original title, name of substance word, subject heading word, floating sub-heading word, keyword heading word, organism supplementary concept word, protocol supplementary concept word, rare disease supplementary concept word, unique identifier, synonyms]

Pubmed:
((severe acute respiratory syndrome) or (SARS) or (SARS-CoV-2) or (COVID-19)) and (coronavirus) and (patient)

Filters: **Journal Article, Human, English**

Search date: 2020/05/04
